# Perceived stress among adolescents as a marker for future mental disorders: A prospective cohort study

**DOI:** 10.1177/1403494821993719

**Published:** 2021-03-01

**Authors:** Louise Lindholdt, Merete Labriola, Johan H. Andersen, Mette-Marie Z. Kjeldsen, Carsten Obel, Thomas Lund

**Affiliations:** 1 Department of Public Health, Aarhus University, Denmark; 2 Research Centre for Youth and Employment, Regional Hospital West Jutland, University Research Clinic, Denmark; 3 NORCE, Norwegian Research Centre, Norway; 4 Department of Occupational Medicine, Danish Ramazzini Centre, University Research Clinic, Regional Hospital West Jutland, Denmark; 5 National Centre for Register-based Research, Aarhus University, Aarhus, Denmark; 6 Department of Public Health, University of Copenhagen, Denmark; 7 Centre for Social Medicine, Frederiksberg and Bispebjerg Hospital, Denmark

**Keywords:** Youth, register data, follow-up, questionnaire, self-rated health

## Abstract

**Aim:**

The mental health problems of adolescents are important in relation to their future health and life course. The aim of this study was to investigate perceived stress in adolescence as a marker for later mental disorders.

**Methods:**

The data consisted of a combination of questionnaire and register data for 11,929 adolescents. Perceived stress was measured using Cohen’s Perceived Stress Scale divided into low, moderate and high perceived stress. Mental disorder was identified using the ICD-10 codes for Mental and Behavioural Disorders classified into whether the adolescents were diagnosed or not. Logistic regression was used to examine the prospective association between perceived stress and mental disorders during about 12 months of follow-up, including the adolescents self-rated health, sex and parental education.

**Results:**

In total, 247 adolescents (2.1%) were diagnosed with a mental disorder during follow-up. The perceived stress of the adolescents was associated with mental disorders, yielding two-fold higher odds of developing a mental disorder for adolescents reporting moderate perceived stress and six-fold higher odds among adolescents reporting high perceived stress in the adjusted model.

**Conclusions:**

**Adolescents with high levels of perceived stress were more likely to develop a mental disorder. Interventions to reduce perceived stress among adolescents could therefore potentially help to identify groups at high risk for later mental disorders.**

## Background

Mental health problems among adolescents are a public health challenge [1] that impacts their daily life course and may have serious consequences on their wider health and life-shaping development [2,3]. Adolescence is a key period and one of the most sensitive phases in an individual’s life, characterised by multiple changes and challenges when evolving from childhood into adulthood [4]. Over the course of adolescence, young people must navigate between trying to find their identity, where they belong and relate to the demands of school, family and friends, as well as choices related to their long-term future in terms of education and employment. This can be a complex and stressful process requiring independent decision-making.

In general, stressful life experiences are considered an important environmental contribution to the risk of developing psychological problems among adolescents [5]. However, it is unknown to what extent adolescents’ find their lives unpredictable, uncontrollable and overloaded, or whether this “carries over” into young adulthood and further. The aim of this study was to investigate perceived stress in adolescence as a marker for later mental disorders.

## Methods

### Data and study population

This prospective cohort study was based on data from the Future Occupation of Children and Adolescents Cohort (the FOCA cohort) [6]. The FOCA cohort is a Danish national youth cohort established in 2017 and consisting of 13,100 adolescents attending the graduating year of compulsory school (ninth grade), independent of school type. Of the 13,100 adolescents, 51.0% were girls and 49.0% were boys, with a mean age of 15.9 years. The adolescents answered a comprehensive online questionnaire and were primarily recruited through their schools. More details about the FOCA cohort and the content of the questionnaire have been described previously [6].

For each individual, data were linked to their unique personal identification number, the CPR-number, which is a 10-digit identification number assigned to all Danish citizens at birth or on immigration [7,8]. This enabled a further linkage to national registers through Statistics Denmark, which provided a unique combination of subjective and objective data on all adolescents. In this study, adolescents were excluded if they had been diagnosed with a preceding mental disorder at the time that they entered the FOCA cohort (*n*=1171), yielding a study population of 11,929 adolescents with no history of mental disorders.

### Measures

#### Adolescents’ mental disorders

Data on adolescents’ mental disorders were obtained from The Danish National Patient Register (DNPR), which contains information on admissions and activities at somatic and psychiatric hospitals [9–11]. For all hospital contacts, the DNPR includes administrative information and information on diagnoses and diagnosis types [9–11].

The administrative data encompass information such as admission type (acute or non-acute contacts), patient contact type (inpatients, outpatients or emergency room patients), referral information, and dates of admission and discharge for each hospital contact. The diagnoses are classified in accordance with the International Classification of Diseases (ICD-10). The ICD-10 codes for Mental and Behavioural Disorders, F00–F99, were applied to identify adolescents’ hospital contacts with a primary diagnosis related to mental disorders, where the date of discharge was used to identify and delimitate diagnoses. In patients with a missing date of discharge, the date of admission was used. Diagnoses related to all patient contact types were included.

Adolescents’ mental disorders were applied as an outcome variable, measured as whether adolescents were diagnosed or not after they answered the FOCA cohort questionnaire during the first quarter of 2017 up until May 2018. Depending on the date of answering the questionnaire, this yielded a follow-up period on all adolescents of at least one year (12–16 months). Mental disorder was applied as a dichotomous variable and no differentiation between diagnoses was made in the main analyses. Similarly, no differentiation was made between adolescents with a single occurrence of mental disorder or repeated events during the follow-up period.

#### Perceived stress

Adolescents’ level of perceived stress (PSS) was measured by the 10-item version of Cohen and colleagues’ Perceived Stress Scale as included in the FOCA cohort questionnaire [12,13]. Each item was rated on a five-point Likert scale ranging from ‘never’ to ‘very often’ (scored 0–4). PSS was calculated as the total sum score (0–40), with higher scores indicating higher levels of PSS (Cronbach’s α=0.8). Single mean imputation was used in patients with missing items on the total scale on <25% equalling less than three items. PSS was used as a categorical variable divided into low, moderate and high PSS based on the tertiles. PSS was applied as an exposure variable.

#### Self-rated health

Self-rated health (SRH) was measured by one item originating from the Short-Form Health Survey (SF-36) on general self-rated health [14], which was incorporated in the FOCA cohort questionnaire. The adolescents assessed their SRH based on the question ‘In general, would you say your health is excellent, very good, good, fair or poor’. SRH was dichotomized in high SRH (‘excellent’ or ‘very good’) or moderate SRH (‘good’, ‘fair’ or ‘poor’) based on a dichotomization used in previous studies [15,16].

#### Parental education

The educational level of the parent with the highest level of completed education was used as a proxy for socioeconomic status. The educational level was obtained from the Danish Education Register of Statistics Denmark [17] and was categorized into four groups based on the years spent on education: <10 years; 10–12 years; 13–15 years; and >15 years [15].

### Statistics

An initial descriptive analysis was made stratified on adolescents with and without mental disorders. A χ^2^- or *t*-test was performed to test for significant differences depending on the type of variable. It was also tested in a descriptive sub-analysis, which mental disorders were the most common with reference to the groups as classified in ICD-10 Chapter V of Mental and Behavioural disorders, F00-F99.

A logistic regression analysis was used to investigate the association between perceived stress among adolescents and later mental disorders. The regression analysis was applied in two steps: an unadjusted crude analysis of all variables’ independent association with adolescents’ mental disorders and a fully adjusted analysis examining adjusted odds ratios (OR) with corresponding 95% confidence intervals (CI) between PSS and mental disorders, adjusted for the adolescents’ SRH, sex and parents’ educational level. A test for interaction between PSS and sex was performed. A correlation analysis of all variables was carried out using Spearman’s rank correlation test. All analyses were performed using the statistical software STATA v. 15.1.

### Ethics approval

The Danish Data Protection Agency and the Ethics Committee of Statistics Denmark approved the FOCA cohort (no. 1-16-02-461-16) in accordance with the rules and legislation on data for research. It was clearly emphasized that the completion of the FOCA cohort questionnaire was anonymous and voluntary to answer, and that all responses would be treated anonymously and strictly confidential. If desired, it was possible to withdraw from participation in the cohort at any time.

## Results

Table I presents descriptive statistics stratified on adolescents’ mental disorders. The characteristics were significantly different between adolescents with and without mental disorders. A total of 247 (2.1%) adolescents were diagnosed with a mental disorder during the follow-up period. Of these, 92.3% were outpatients.

**Table I.** Characteristics of the study population.

Characteristic	Adolescents without mental disorders (*N* = 11,682)	Adolescents with mental disorders (*N* = 247)	*P*
Sex			<0.001^a^
Boys	5724 (49.0)	85 (34.4)	
Girls	5958 (51.0)	162 (65.6)	
Age (years)	15.8 ± 0.4	15.9 ± 0.5	0.02^b^
Parents’ educational level (years)			0.02^a^
<10	593 (5.1)	22 (8.9)	
10–12	4767 (40.8)	113 (45.7)	
13–15	4209 (36.0)	76 (30.8)	
>15	1878 (16.1)	31 (12.6)	
Missing data	235 (2.0)	5 (2.0)	
General self-rated health			<0.001^a^
High	7358 (63.0)	83 (33.6)	
Moderate	2739 (23.4)	134 (54.3)	
Missing data	1585 (13.6)	30 (12.1)	
Perceived Stress Scale			<0.001^a^
Low perceived stress	3453 (29.6)	18 (7.3)	
Moderate perceived stress	3532 (30.2)	49 (19.8)	
High perceived stress	2932 (25.1)	147 (59.5)	
Missing data	1765 (15.1)	33 (13.4)	

Data presented as no. (%) or mean ± SD values.

a χ^2^ test.

b *t*-test.

Adolescents who were diagnosed with a mental disorder reported higher levels of PSS than adolescents who were not diagnosed with a mental disorder (Table I). For adolescents diagnosed with a mental disorder, 7.3% reported low PSS, 19.8% reported moderate PSS and 59.5% reported high PSS. The corresponding distribution for adolescents who were not diagnosed with a mental disorder was 29.6% with low PSS, 30.2% with moderate PSS and 25.1% with high PSS. Similarly, SRH was lower among adolescents who were later diagnosed with a mental disorder.

The additional analysis revealed that among the 247 adolescents who were diagnosed with a mental disorder during the follow-up period, 40% of the diagnoses were within DF40–48 (neurotic, stress-related and somatoform disorders), constituting the largest share among the subgroups of mental disorders as classified in ICD-10 (analysis not shown). The 247 unique adolescents constituted 412 observations of mental disorders because some adolescents were diagnosed more than once.

The regression analysis revealed that adolescents’ level of PSS was significantly associated with mental disorders during the follow-up period, showing an unadjusted OR of 2.7 (95% CI 1.6–4.6) for adolescents with moderate PSS and OR 9.6 (95% CI 5.9–15.7) for adolescents reporting high PSS compared with adolescents with low PSS (Table II). The association remained in the adjusted analysis considering the adolescents’ SRH, sex and parents’ educational level with an adjusted OR of 2.2 (95% CI 1.3–3.8) for adolescents reporting moderate PSS and an adjusted OR of 6.1 (95% CI 3.6–10.1) for adolescents with high PSS. Adolescents with moderate SRH had higher odds for being diagnosed with a mental disorder compared with adolescents with high SRH, yielding an adjusted OR of 2.6 (95% CI 2.0–3.5) for adolescents with moderate SRH. Higher odds for mental disorders were found among girls compared to boys in the unadjusted model, but this was attenuated in the adjusted model. The importance of parental educational level was similar in both analyses. A test for interaction between PSS and sex was made, but no interaction was found.

**Table II.** Odds ratios for mental and behavioural disorders after completing the questionnaire.

	Unadjusted	Adjusted
Perceived Stress Scale		
Low perceived stress	1.0 (ref.)	1.0 (ref.)
Moderate perceived stress	2.7 (1.6–4.6)	2.2 (1.3–3.8)
High perceived stress	9.6 (5.9–15.7)	6.1 (3.6–10.1)
Self-rated health		
High	1.0 (ref.)	1.0 (ref.)
Moderate	4.3 (3.3–5.7)	2.6 (2.0–3.5)
Sex		
Boys	1.0 (ref.)	1.0 (ref.)
Girls	1.8 (1.4–2.4)	1.4 (1.0–1.9)
Parents’ educational level (years)		
>15	1.0 (ref.)	1.0 (ref.)
13–15	1.1 (0.7–1.7)	1.1 (0.7–1.7)
10–12	1.4 (1.0–2.1)	1.3 (0.8–1.9)
<10	2.3 (1.3–3.9)	1.8 (1.0–3.2)

Data presented as odds ratios (95% confidence interval).

The sample size of the adjusted model consisted of 9949 adolescents. A non-responder analysis on PSS (*n*=1798) did not indicate significant differences between adolescent responders and non-responders (results not presented). The correlation analysis between all variables showed no correlation higher than a Spearman’s ρ of 0.3 (results not presented).

## Discussion

An association was found between adolescents’ PSS and later mental disorders. After controlling for SRH, sex and parents’ educational level, the study found two-fold higher odds of being diagnosed with a mental disorder among adolescents with moderate PSS and six-fold higher odds among adolescents with a high level of PSS compared with adolescents with low PSS. Also, moderate SRH more than doubled the odds of experiencing a mental disorder compared with high SRH in the adjusted model, which indicated confounding by SRH.

This is in line with previous findings within the field of perceived stress and mental health. Bovier et al. [18] found stress and internal resources as strong correlates with mental health in a study of 1257 university students, with stress being of particular importance, explaining more than half of the total variance in mental health. A study by Schraml et al. [19] found that those adolescents who experienced the most stress symptoms were more likely to feel worse about themselves, as well as having more sleep problems than non-stressed adolescents. Related studies found a relationship between adolescent stress and later depressive symptoms. However, these studies focused on different aspects of mental health and psychological well-being, specific mental disorders or applied different measures for assessing perceived stress [18,20–22].

The use of both subjective and objective measures of health-related conditions is considered a strength because it contributes to varied and nuanced information. PSS and SRH are validated measures contributing to a subjectively assessed specific knowledge of adolescents’ PSS in different situations and their state of health [13,14]. In this study, the Cronbach’s α of PSS was 0.8, indicating a good internal consistency in line with Cohen et al. [12]. Despite SRH being a measure of general health and previously found to be a strong predictor for mortality and morbidity [23,24], adolescents tend to strongly emphasize their mental state of health when rating their SRH [23], for which reason it seemed relevant to include this when studying adolescent mental disorders. Although self-reported measures can lead to inaccuracies and a risk of information bias, the information used seemed to be acceptable.

Information on mental disorders stemmed from a comprehensive register [8,9]. The use of registry data contributed with no loss to follow-up on mental disorders and therefore reduced the risk of bias. However, only adolescents diagnosed with mental disorders through a hospital contact (inpatients, outpatients or emergency room patients) were included and therefore, to some extent, represented a selected patient group of adolescents potentially characterized by a higher severity of the disease in question [9]. As the DNPR only covers diagnoses related to a hospital contact [11], undiagnosed mental health problems and milder or moderate cases treated by general practitioners, which in Denmark is true for many mild to moderate mental disorders [11], were not included in this study. This could lead to an underestimation of the findings, although it did not impact the associations presented. It is not possible to rule out whether the adolescents felt mentally unwell before answering the questionnaire because some time will pass before they are diagnosed and referred for psychiatric treatment.

Adolescents who had previously been diagnosed with mental disorders were excluded to give a homogeneous study population. Excluding these adolescents could influence the findings and potentially cause an underestimation, which is also the case with non-responders. Prior cases of mental disorders could potentially have a modifying effect, but this was not tested.

This study did not analyse specific diagnoses in the main analysis, but overall mental disorders. However, the additional descriptive analysis revealed that a large percentage of the adolescents were diagnosed within DF40–48 (neurotic, stress-related and somatoform disorders). Further research could focus on the association with specific diagnoses when analysing PSS and SRH. Moreover, as few cases were diagnosed during the approximately one-year follow-up period, it would be desirable to replicate this study with an extended follow-up period. As 13.1% of adolescents diagnosed with a mental disorder had missing data on PSS, this supports the need for replicating the study on a larger population of adolescents.

## Conclusions

To the best of our knowledge, this study is the first to investigate the association of PSS levels using the 10-item Perceived Stress Scale of Cohen et al. [12] and later mental disorders among an adolescent population. Adolescents’ PSS can be of great importance for suggesting later mental disorders, which could benefit future research in studies of adolescents’ further life course. PSS can potentially aid in identifying adolescent sub-populations at high risk of developing severe future mental health problems. From a public health point of view, further research is needed and the potential consequences of PSS and mental disorders during such a sensitive and critical life phase as adolescence, with further investigations of their educational and working life trajectories, would be important.
